# Age-related CCL12 Aggravates Intracerebral Hemorrhage-induced Brain Injury via Recruitment of Macrophages and T Lymphocytes

**DOI:** 10.14336/AD.2019.1229

**Published:** 2020-10-01

**Authors:** Jiacheng Huang, Guoqiang Yang, Xiaoyi Xiong, Maolin Wang, Junjie Yuan, Qin Zhang, Changxiong Gong, Zhongming Qiu, Zhaoyou Meng, Rui Xu, Qiong Chen, Ru Chen, Lexing Xie, Qi Xie, Wenjie Zi, Guohui Jiang, Yu Zhou, Qingwu Yang

**Affiliations:** ^1^Department of Neurology, Xinqiao Hospital, the Army Medical University (Third Military Medical University), Chongqing 400037, China.; ^1^Central Laboratory, Xinqiao Hospital, the Army Medical University (Third Military Medical University), Chongqing 400037, China.

**Keywords:** age, CCL12, intracerebral hemorrhage, inflammation, parabiosis

## Abstract

Circulating factors associated with aging have been shown to be involved in the development of age-related chronic and acute brain diseases. Here, we aimed to investigate the roles and mechanisms of CCL12, a circulating factor that is highly expressed in the plasma of aged rodents after intracerebral hemorrhage (ICH) using parabiosis and ICH models. Neurological deficit score (NDS), mortality rate, brain water content (BWC), and levels of inflammatory factors were determined to assess the degree of ICH-induced brain injury. Peripheral inflammatory cell infiltration was examined using immunofluorescence and flow cytometry. After confirming that acute brain injury after ICH was aggravated with age, we found that brain and plasma CCL12 levels were markedly higher in old mice than in young mice after ICH, and that plasma CCL12 was able to enter the brain. Using CCL12^-/-^ mice, we showed that the degree of damage in the brain—as determined by NDS, mortality rate, BWC, levels of inflammatory factors, and numbers of degenerative and apoptotic neural cells and surviving neurons was significantly attenuated compared to that observed in old wild-type (WT) mice. These effects were reversed in CCL12-treated old mice. The detrimental effects caused by CCL12 may involve its ability to recruit macrophages and T cells. Finally, the administration of an anti-CCL12 antibody markedly improved the outcomes of ICH mice. Our results are the first to indicate that elevated peripheral CCL12 levels in old mice aggravates ICH-induced brain injury by recruiting macrophages and T cells. Thus, CCL12 may be a new target for ICH treatment.

Intracerebral hemorrhage (ICH) is a common disorder with high mortality and morbidity, especially in China [1-3]. Generally, elderly patients with ICH have a worse prognosis than young patients, indicating that age is a major factor that aggravates brain injury [4]. Therefore, elucidating the mechanisms by which aging aggravates brain injury has great therapeutic significance for ICH.

Intriguingly, we and others have shown that transplanting plasma from young individuals can improve brain biofunction in old mice and even ameliorate acute brain injury in old rodents with ICH and ischemic stroke [5, 6]. These results strongly suggest that circulating factors can significantly influence the process of normal aging in the brain and diseased brain. The levels of circulating factors, such as CCL2, CCL11, CCL12, CCL19, haptoglobin and β2-microglobulin are markedly elevated in old mice [7]. Among them, CCL2, CCL11, CCL19, haptoglobin and β2-microglobulin have been shown to be significantly involved in the progression of normal aging and age-related acute brain injuries [8-11]. However, whether elevated plasma CCL12 concentrations in old mice are also correlated with acute brain injury after ICH remains unclear.

CCL12, also known as monocyte chemotactic protein (MCP-5), is a chemokine that plays an important role in the inflammatory response, as it recruits peripheral immune cells to damaged areas [12]. Therefore, the goal of this study was to investigate whether CCL12 can aggravate ICH-induced brain injury by recruiting peripheral immune cells. We found that peripheral CCL12 aggravated ICH-induced brain injury by recruiting macrophages and T cells and that the systemic administration of an anti-CCL12 antibody improved the outcomes in old ICH mice. Thus, peripheral CCL12 may be considered a novel therapeutic target for ICH treatment.

## MATERIALS AND METHODS

### Animals

Young C57BL/6 mice (male, 8 weeks old, 18-24 g) and adult mice (12 months old) were obtained from the Animal Center of the Army Medical University (Chongqing, China). The old mice were obtained when they were 12 months old and were maintained in our animal center for at least 6 months until they were 18-20 months old. CCL12^-/-^ mice were purchased from Jackson Laboratory (Bar Harbor, ME, USA). The old CCL12^-/-^ mice were maintained until they were at least 18 months old. The mice were housed in specific pathogen-free grade animal rooms under constant temperature and appropriate lighting conditions. The mice were given free access to food and water. The mice were randomly divided into groups and the investigators were blinded to the group information.

### Parabiosis

Male mice with similar weights and sizes were first housed together for 2-3 weeks before surgery, and the procedures were performed as described previously [13]. Wild-type (WT) mice were used as parabiotic donors of CCL12 to CCL12^-/-^ mice. ICH was induced in the WT or CCL12^-/-^ parabionts at least 2 weeks after parabiotic surgery [14].

### ICH model

The ICH model was developed as described previously [15]. Briefly, the mice were anesthetized with isoflurane at 3% for induction and 1.5% for maintenance of anesthesia. Then the mice were immobilized on a stereotaxic apparatus (RWD Life Science Co., Shenzhen, China). A total of 20 μl of whole autologous blood or the same volume of saline was injected at 2.5 μl/min into the striatum (0.8 mm anterior and 2 mm lateral to bregma and at a depth of 3.5 mm). The failed models and mice that died were excluded.

### Fluorescence-activated cell sorting (FACS) analysis

Single-cell suspensions of brain cells were prepared as described previously [16, 17]. Briefly, mice (n = 6) were perfused using ice-cold phosphate-buffered solution (PBS) to clear blood cells. Each hemorrhagic hemisphere was digested in a rotating shaker at 37? for 45 min after the addition of RPMI Medium 1640 (Solarbio, Beijing, China) supplemented with 1 mg/ml collagenase Ⅳ (Sigma-Aldrich, St. Louis, MO, USA) and 100 U/ml DNase I (Sigma-Aldrich). Then, double the volume of 1640 RPMI containing 10% fetal bovine serum was added to stop the digestion. After centrifugation, the supernatant was discarded and the cells were resuspended in 37% Percoll (GE Healthcare, Pittsburgh, PA, USA). Then, the cell suspension was transferred onto 70% Percoll and centrifuged at 400× *g* for 25 min. The interlayer between the two gradients was collected, and the cells were stained with CD45-BV421 (1:200; BioLegend, San Diego, CA, USA), CD11b-PE/Cy7 (1:200; BioLegend), CD3-APC (1:200; BioLegend), CD19-FITC (1:200; BioLegend), and Ly6G-PerCP (1:200; BioLegend) antibodies at 4?for 20 min in the dark. All the samples were analyzed using a FACSVerse analyzer (BD, Franklin Lakes, NJ, USA). All the results were analyzed using FlowJo 7.6.1.

### CCL12 protein and antibody administration

Carrier-free recombinant murine CCL12 dissolved in PBS (10 μl/kg; R&D Systems, Minneapolis, MN, USA) and a rat IgG2a neutralizing antibody against mouse CCL12 (50 μl/kg; R&D Systems) were systemically administered via intraperitoneal injection 1 h before ICH surgery.

### Neurological deficit scores (NDS) assessment

A 28-potint NDS assessment system was adopted[18]. Climbing, front limb symmetry, circling behavior, and body symmetry were scored by three trained investigators who were blinded to the group information. The mean of the scores was used as the final score for each mouse.

### Survival analysis

The number of mice that died was recorded on days 1, 3, 5, and 7 after ICH for survival analysis. The survival rate was calculated as follows: (the number of ICH mice per group - the number of dead mice per group)/the number of ICH mice per group.

### Brain water content (BWC) measurement

As described in our previous study [19], one day after the ICH procedure, the mice were anesthetized by intraperitoneal injection, and the ipsilateral brain tissues were removed. The samples were divided into three portions: the ipsilateral, contralateral and cerebellum portions. First, the water on the surface of the tissues was wiped with filter paper before the tissues were weighed. Next, after the brain tissues were dried at 100 ? for an entire day, the dry weight was measured. The BWC was calculated using the following formula: (%) = (wet weight - dry weight)/wet weight × 100%.

### Quantitative real-time PCR

Real-time PCR was performed according to the manufacturer’s instructions (Takara Biotechnology, Dalian, China). Glyceraldehyde 3-phosphate dehydrogenase (GAPDH) was used as an internal control, and the relative levels of mRNA expression were calculated using the 2^-△△CT^ formula. The primers used for real-time PCR are shown in Table 1.

**Table 1:** Primers used for quantitative real-time PCR.

Gene	Forward primer	Reverse primer
*CCL12*	5′-ATTTCCACACTT CTATGCCTCCT-3′	5′-ATCCAGTATGGT CCTGAAGATCA-3′
*GAPDH*	5′-GGTTGTCTC CTGCGACTTCA-3′	5′-TGGTCCAGGGT TTCTTACTCC-3′

### Western blot

As described in our previous study [20], proteins extracted from perihematomal tissues were resolved by sodium dodecyl sulfate polyacrylamide gel electrophoresis (SDS-PAGE) and transferred onto polyvinylidene fluoride membranes by electroblotting. Subsequently, the membranes were incubated with a rabbit anti-mouse CCL12 antibody (1:400; LSBio, Seattle, WA, USA) at 4?. The membranes were then incubated with HRP-conjugated goat anti-rabbit secondary antibodies (1:5000; Millipore, Boston, MA, USA) at room temperature for 1.5 h. Bound antibodies were observed using a chemi-luminescence detection system. Signals were quantified by scanning densitometry and computer-assisted image analysis. GAPDH was used as a loading control. Protein levels were expressed as the ratio of the value of the detected protein band to that of the GAPDH band.

### Enzyme-linked immunosorbent assay (ELISA)

Eyeball blood samples were collected from euthanized mice and were allowed to clot for 2 h at room temperature before being centrifugated for 20 min at 2000 × *g*. Serum CCL12 levels were detected using a mouse CCL12 ELISA kit (R&D Systems).Serum IL-6, IL-10, and tumor necrosis factor (TNF-α) levels were also measured using ELISA kits (CUSBIO, Wuhan, China).

### Hematoxylin and eosin (H&E) staining

Following our previously described method[13], brain tissues were removed from euthanized mice and fixed in 4% paraformaldehyde. Next, the samples were dehydrated in a series of graded ethanol solutions and then embedded in paraffin for sectioning with a rotary microtome into 4.5-μm-thick slices. Finally, the slides were stained with H&E and subsequently observed using an Olympus microscope.

### Fluoro-Jade B (FJB) staining

FJB staining was performed as described in our previous study [21]. Briefly, brain tissues were dehydrated in 30% sucrose solution and cut into 10-μm-thick sections. Next, the sections were immersed in 1% sodium hydroxide in 80% alcohol for 10 min, followed by incubation in 70% alcohol for 2 min and distilled water for 5 min. Subsequently, the sections were incubated in 0.06% potassium permanganate for 10 min at room temperature and then washed in distilled water. Then, the sections were incubated in a 0.01% FJB solution (Millipore) for 30 min and washed with distilled water. Next, the sections were dehydrated in gradient alcohol solutions, washed in xylene and covered with coverslips with DPX (Sigma-Aldrich). The FJB-stained tissue sections were visualized under an Olympus microscope. Positive cells around the hematoma were counted, and the number of positive cells in each standardized microscope field was analyzed dependently by three researchers using ImageJ (version 1.46J, National Institute of Health, Bethesda, MA, USA). The average value of 3 regions of interest was used as the final value.

### Terminal deoxynucleotidyl transferase dUTP nick end labeling (TUNEL)

A commercial*In Situ* Cell Death Detection kit (Roche, Basel, Switzerland) was used to detect neuronal apoptosis according to the manufacturer’s instructions. For each group, the positive cells in three randomly chosen high-power fields (400×) around the hematoma area were counted using a light microscope. The number of positive cells in each standardized microscopic field was analyzed independently by three researchers using ImageJ (version 1.46J). The average value of three regions of interest was used as the final value.

**Figure 1.**
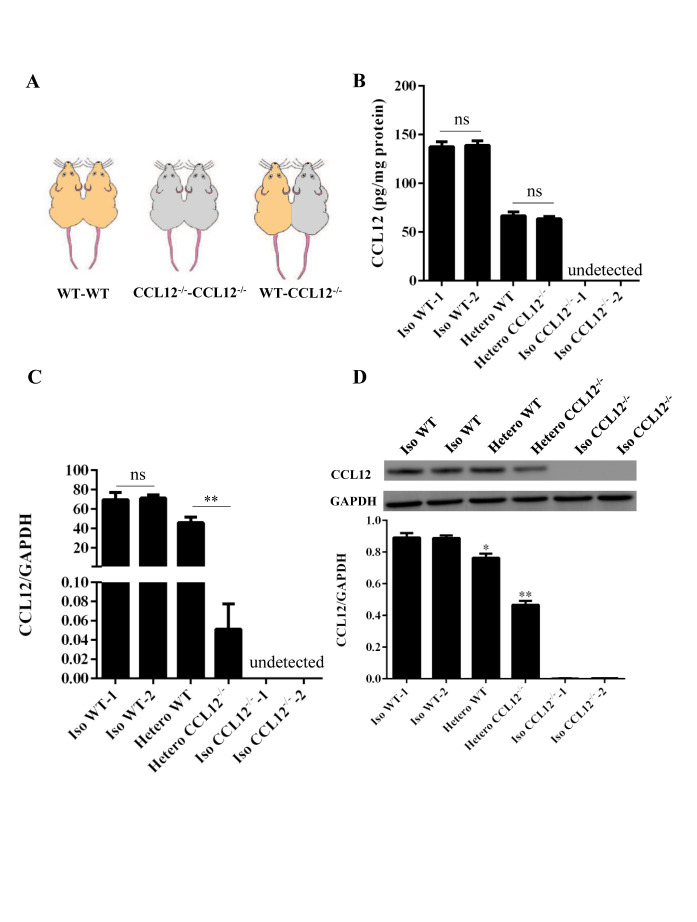
Peripheral CCL12 can enter the brain after ICH. (A) A schematic showing the parabiotic pairings. (B) Peripheral CCL12 protein was detected by ELISA in isochronic WT, heterochronic WT and CCL12^-/-^, and isochronic CCL12^-/-^ mice. (C) CCL12 mRNA expression was detected in the brain of isochronic (WT-WT and CCL12^-/-^-CCL12^-/-^) and heterochronic (WT-CCL12^-/-^) mice after ICH by real-time PCR. (D) CCL12 protein expression was detected by western blot in the perihematomal brain tissues from isochronic WT and heterochronic mice after ICH. The bar graphs show the means ± SDs. n = 5. *P*-values were determined by ANOVA. *P< 0.05; **P< 0.01; ns, not significant.

### Nissl staining

Nissl staining was performed according to our previously described method [21]. Briefly, brain tissue sections were stained with thionin (Sigma-Aldrich), dehydrated in alcohol, cleared in xylene, and then sealed with coverslips to allow the perihematomal brain tissues to be observed. For the quantification of the Nissl staining results, the number of cells in each standardized microscope field was analyzed independently by three researchers using ImageJ (version 1.46J). The average value of three regions of interest was used as the final value.

### Immunofluorescence

Immunofluorescence was performed according to our previously described method [16]. Brain tissues were resected from the mice, fixed in 4% paraformaldehyde for 24 h, dehydrated with 30% sucrose solutions, and frozen in compound. The sections (40-μm thick) were blocked with 0.1% Triton X-100 and 5% normal donkey serum in PBS for 20 min, and then incubated with the following primary antibodies overnight at 4°C: anti-F4/80 (1:200; Abcam, Cambridge, UK) and anti-CD3 (1:200, Abcam). The sections were then incubated with a mixture of Alexa Fluor 647-conjugated goat anti-rat IgG (1:1000; Thermo Fisher Scientific, Waltham, MA, USA) and Alexa Fluor 488-conjugated goat anti-rabbit IgG (1:1000; Thermo Fisher Scientific) antibodies for 30 min at 37°C. Next, 4′,6-diamidino-2-phenylindole (DAPI, 1:3000; Sigma-Aldrich) was applied for 5 min, after which the samples were washed three times in PBS for 15 min. The sections were then mounted on SuperFrost slides, and all images were captured using a confocal fluorescence microscope (TCS-TIV; Leica, Nussloch, Germany).

### Immunohistochemistry

Formalin-fixed paraffin-embedded brain tissues were sectioned into 4.5-µm-thick slides. Ly6G single-stain immunohistochemistry was performed using an anti-neutrophil elastase antibody (1:200; Abcam) following the manufacturer’s instructions, after which the perihematomal brain tissues were observed. For the quantification of neutrophils, the number of cells in each standardized microscope field was analyzed independently by three researchers using ImageJ (version 1.46J). The average value of three regions of interest was used as the final value.

**Figure 2.**
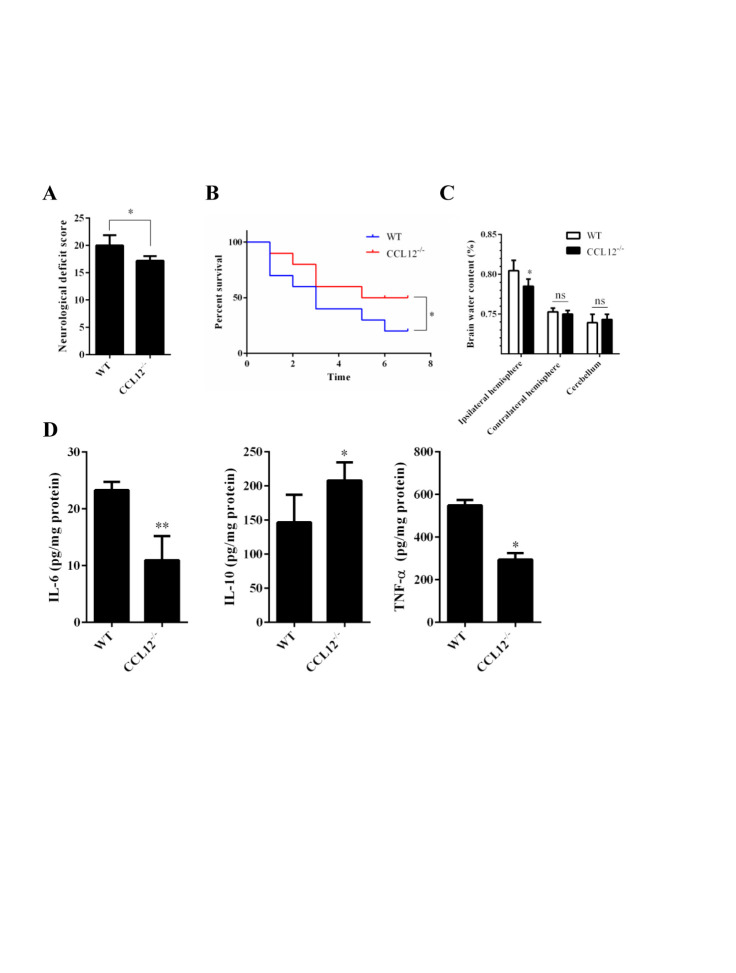
CCL12 knockout increases inflammatory injury in perihematomal brain tissues after ICH. (A-C) NDS (n = 5), mortality rate (B) (n = 20) and BWC (C) (n = 5) in WT and CCL12^-/-^ mice after ICH. (D) IL-6, IL-10 and TNF-α protein levels in WT and CCL12^-/-^ mice detected by ELISA after ICH (n = 6). The bar graphs show the means ± SDs.*P*-values were determined by ANOVA. *P< 0.05; **P< 0.01; ns, not significant.

### Statistical analysis

The data are expressed as the means ± standard deviations (SDs) or as percentages. Statistical differences between pairs of groups were assessed using the unpaired *t*test and among multiple groups using two-way analysis of variance ANOVA. Kaplan-Meier survival analysis was used to compare the survival rates. Differences were considered significant at *P*< 0.05.

## RESULTS

### Old mice have worse outcomes than young mice after ICH

To evaluate the effects of aging on the prognosis of ICH, young and old mice underwent ICH surgery and were euthanized 1 d after ICH because many proinflammatory cytokines reach their maximum levels at this time [22, 23]. The results showed that the NDS, mortality rate, BWC of perihematomal brain tissues and levels of inflammatory cytokines, such as IL-6, IL-10 and TNF-α, were lower in old mice than in young mice after ICH (Supplementary Fig. 1), which was consistent with our previous research [13].

### Brain and plasma CCL12 levels increase markedly with age in ICH mice

Previous studies have reported that levels of the circulating factor CCL12 are markedly higher in old mice than in young mice, and that this rise may be involved in the progression of brain injury caused by ICH [7]. Therefore, before investigating the roles of circulating CCL12 in acute brain injury, we first examined the expression levels of brain and plasma CCL12 in young and old mice with or without ICH using ELISA and real-time PCR. Plasma CCL12 levels in normal old mice were markedly elevated (Supplementary Fig. 2B), which was similar to previously reported results[7]. When the mice were subjected to ICH, the plasma CCL12 levels significantly increased, and more so in old mice than in young mice (Supplementary Fig. 2B). In addition, CCL12 expression in perihematomal brain tissues was also significantly increased in old ICH mice (Supplementary Fig. 2A), with both CCL12 mRNA (Supplementary Fig. 2C) and protein (Supplementary Fig. 2D) levels peaking 1 d after ICH.

**Figure 3.**
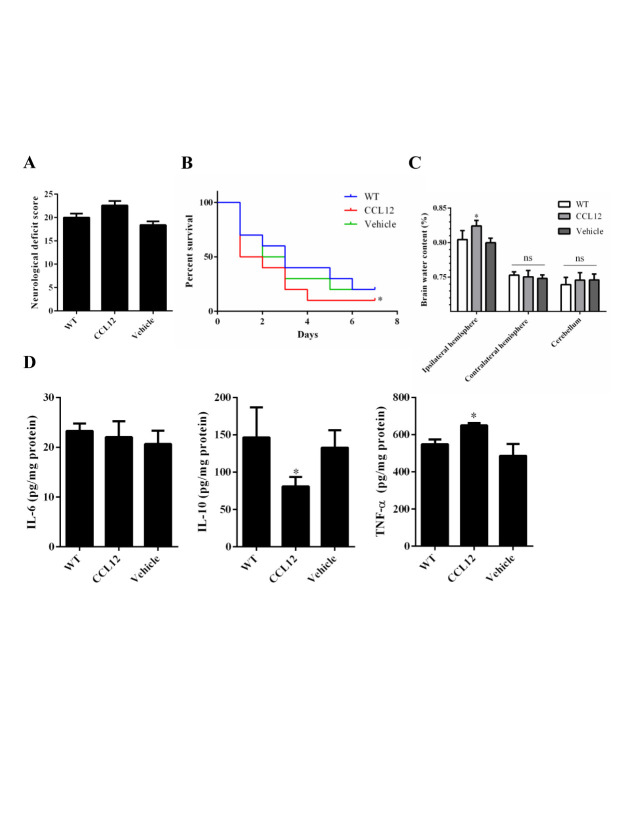
CCL12 supplementation aggravates ICH-induced brain injury in mice. (A-C) NDS (n = 5), mortality rate (B) (n = 20) and BWC (n = 5) levels of WT, old CCL12-treated and vehicle-treated mice after ICH. (D) IL-6, IL-10 and TNF-α protein levels in WT, old CCL12-treated and vehicle-treated mice were detected by ELISA after ICH (n = 6). The bar graphs show the means ± SDs. n = 5.*P*-values were determined by ANOVA. *P< 0.05; **P< 0.01; ns, not significant.

**Figure 4.**
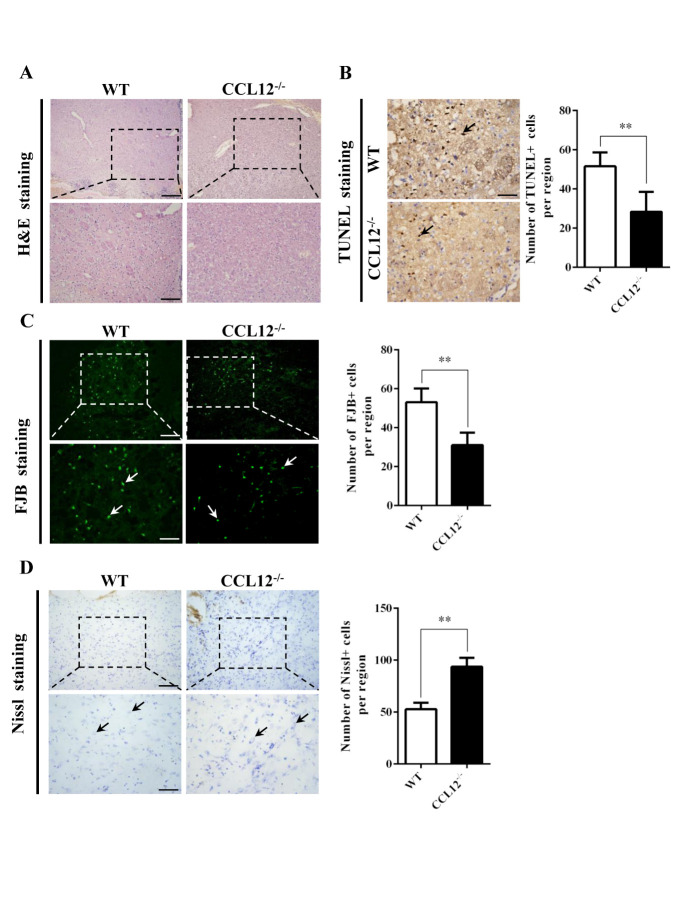
CCL12 knockout increases the number of surviving neurons. (A) H&E staining of perihematomal brain tissues from old WT and CCL12^-/-^mice after ICH surgery. Scale bars, 200 μm top images and 100 μm bottom images. (B) TUNEL assay results and quantification of apoptotic cells in perihematomal brain tissues derived from WT and CCL12^-/-^ mice after ICH. Scale bars, 100 μm. (C) FJB staining and quantification of degenerative cells in perihematomal brain tissues derived from WT and CCL12^-/-^ mice after ICH surgery. (D) Nissl staining and quantification of Nissl bodies in perihematomal brain tissues derived from WT and CCL12^-/-^ mice after ICH. Scale bars, 200 μm on the top and 100 μm on the bottom. Scale bars, 200 μm top images and 100 μm bottom images. The bar graphs show the means ± SDs. n = 5.*P*-values were determined by ANOVA. *P< 0.05; **P< 0.01; ns, not significant.

### Peripheral CCL12 can enter the brain of mice

Next, we determined whether the increased circulating CCL12 could enter the brain and become involved in the brain injury caused by ICH. Following our previous methods [18], we constructed parabiosis models using WT and CCL12^-/-^ mice (Fig. 1A). After 15 d of parabiosis, the circulation of the parabionts was demonstrated to be successfully connected, because circulating CCL12 was detected in the CCL12^-/-^ mice after exposure to WT circulation (Fig. 1B). Next, we evaluated brain CCL12 mRNA and protein levels, and showed that CCL12 mRNA was almost absent in the brain tissues of heterochronic CCL12^-/-^ ICH mice, as assessed by real-time PCR (Fig. 1C). Furthermore, CCL12 protein was detected in the brain tissues of heterochronic CCL12^-/-^ mice by western blot (Fig. 1D). Taken together, these results strongly suggest that circulating CCL12 can enter the brain.

**Figure 5.**
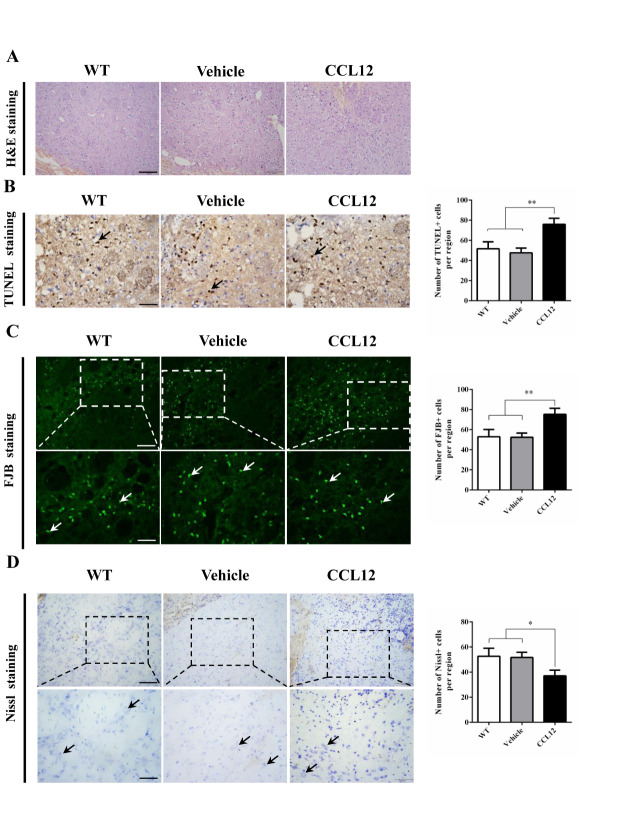
CCL12 treatment decreases the number of surviving neurons. (A) H&E staining of perihematomal brain tissues from old WT, vehicle-treated and CCL12-treated mice after ICH surgery. Scale bars, 200 μm top images and 100 μm bottom images. (B) TUNEL assay results and quantification of apoptotic cells in perihematomal brain tissues derived from old WT, vehicle-treated and CCL12-treated mice after ICH surgery. Scale bars, 100 μm. (C) FJB staining and quantification of degenerative cells in perihematomal brain tissues derived from old WT, vehicle-treated and CCL12-treated mice after ICH surgery. (D) Nissl staining and quantification of Nissl bodies in perihematomal brain tissues derived from old WT, vehicle-treated and CCL12-treated mice after ICH surgery. Scale bars, 200 μm top images and 100 μm bottom images. The bar graphs show the means ± SDs. n = 5.*P*-values were determined by ANOVA. *P< 0.05; **P< 0.01; ns, not significant.

**Figure 6.**
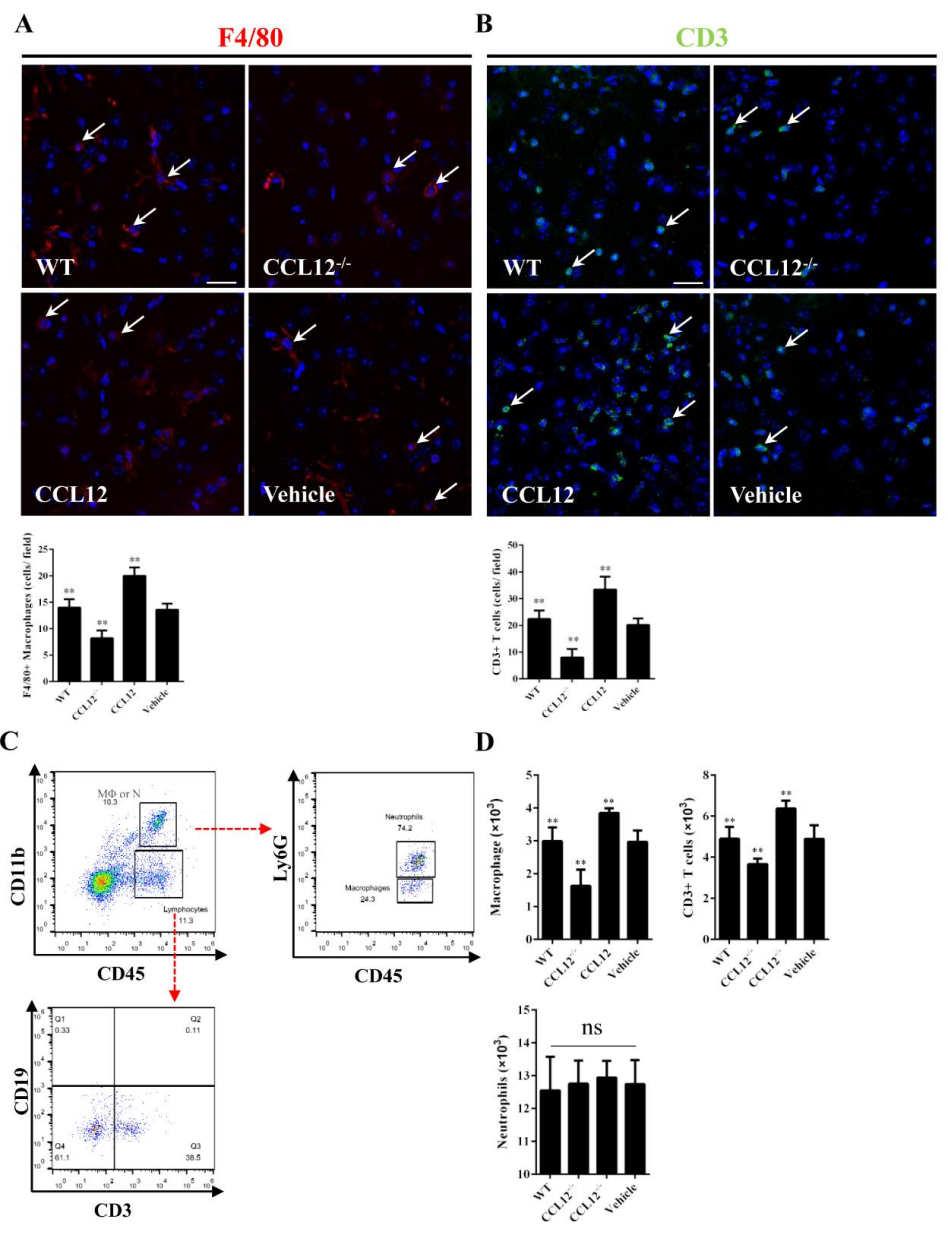
Peripheral CCL12 aggravates the ICH-induced damage by recruiting peripheral macrophages and CD3+ T lymphocytes. (A) Representative fluorescence microscopy images show infiltrating F4/80+ cells (macrophages) in the perihematomal tissues (the arrows indicate F4/80+ cells; blue = 4°-6-diamidino-2-phenylindole [DAPI] and red = F4/80). (B) Representative fluorescence microscopy images show infiltrating CD3+ cells (T lymphocytes) in the perihematomal tissues (the arrows indicate CD3+ cells; blue = DAPI, and green = CD3). (C) CD45+ CD11b+ cells were defined as macrophages and neutrophils and further analyzed to distinguish the two types of cells. (D) The number of macrophages, CD3+ T cells and neutrophils in WT, CCL12^-/-^, CCL12 overexpression, and vehicle groups after ICH. Scale bars, 50 μm. The bar graphs show the means ± SDs.*P*-values were determined by ANOVA. *P< 0.05; **P< 0.01; ns, not significant.

### CCL12 knockout alleviates brain injury in old mice after ICH

After assessing the CCL12 expression profiles in mice after ICH, we then investigated the roles of CCL12 in regulating brain injury in mice after ICH. Compared to old WT mice, old CCL12^-/-^ mice exhibited markedly lower NDS (Fig. 2A) and mortality rate (Fig. 2B) after ICH, which may have been correlated with the reduced BWC observed in old CCL12^-/-^ mice (Fig. 2C). Additionally, compared to old WT ICH mice, old CCL12^-/-^ mice had significantly lower concentrations of IL-6 and TNF-α but higher concentrations of IL-10 after ICH (Fig. 2D). Furthermore, the H&E staining result showed that the number of infiltrated peripheral immune cells around the hematoma area was significantly lower in old CCL12^-/-^ mice than in old WT mice after ICH (Fig. 3A). In addition, the TUNEL and FJB staining results showed that the number of apoptotic (Fig. 3B) and degenerative cells (Fig. 3C) was markedly decreased in old CCL12^-/-^ ICH mice compared to old WT mice after ICH. In contrast, the number of surviving neurons (Fig. 3D) in the perihematomal brain tissues of old ICH CCL12^-/-^ mice was significantly increased. These results indicate that CCL12 deficiency could significantly alleviate ICH-induced brain injury in the ICH mouse model.

### CCL12 supplementation aggravates ICH-induced brain injury in mice

To further confirm the role of CCL12 in ICH-induced brain injury, recombinant CCL12 or vehicle was administered systemically via intraperitoneal injection 1 h before ICH surgery. The systemic administration of CCL12 induced a marked increase in plasma and brain CCL12 protein levels (Supplementary Fig. 3A and 3B). One day after ICH, the mortality rate and BWC in these mice were significantly increased compared to those observed in old WT and CCL12^-/-^ mice (Fig. 3B, C), but no significant differences in the NDS were observed (Fig. 3A). Next, we measured the levels of inflammatory cytokines, and showed that old CCL12-treated mice had higher TNF-α and lower IL-10 levels than control mice, however IL-6 expression was not significantly different between the two groups (Fig. 3D). In contrast to the results obtained from CCL12^-/-^ and vehicle-treated mice, CCL12 supplementation increased the number of infiltrated peripheral immune cells (Fig. 5A), apoptotic cells (Fig. 5B) and degenerative cells (Fig. 5C) around the hematoma areas in old mice after ICH. As expected, the number of surviving neurons (Fig. 5D) in the perihematomal brain tissues of CCL12-treated old mice after ICH was significantly reduced. Thus, these results strongly suggest that CCL12 can significantly aggravate ICH-induced brain injury.

**Figure 7.**
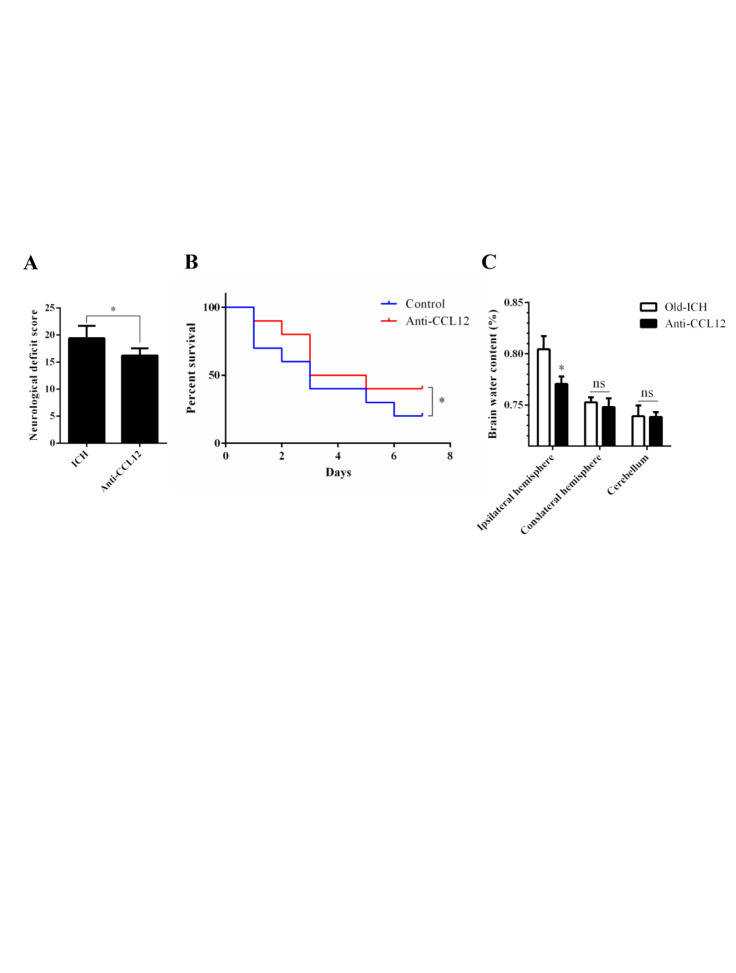
CCL12 antibody treatment improves the prognosis of old mice after ICH. (A-C) NDS (n = 5), mortality rate (B) (n =20) and BWC (C) (n = 5) of old mice treated with an anti-CCL12 antibody after ICH. The bar graphs show the means ± SDs.*P*-values were determined by ANOVA. *P< 0.05; **P< 0.01; ns, not significant.

### CCL12 recruits peripheral immune cells into damaged brain tissues after ICH

Increasing evidence has shown that CCL12 plays an essential role in exacerbating the inflammatory response via recruiting peripheral immune cells to damaged areas [12]. In this study, we first used immunofluorescence and immunohistochemistry to investigate whether elevated CCL12 levels in the brain could promote the infiltration of peripheral immune cells into the brain of old ICH mice. The results showed that fewer F4/80+ macrophages infiltrated the perihematomal brain tissues in old CCL12^-/-^ mice than in old WT mice after ICH, whereas more macrophages were recruited in old CCL12-treated mice (Fig. 6A). Similarly, fewer CD3+ T lymphocytes infiltrated the perihematomal areas in old CCL12^-/-^ mice than in WT mice, while more CD3+ T cells were recruited in old CCL12-treated mice (Fig. 6B). However, no differences in the number of neutrophils were observed between old WT, old CCL12^-/-^, old CCL12-treated and vehicle-treated mice after ICH (Supplementary Fig. 4A). Furthermore, the changes in the numbers of different inflammatory cells in the hemorrhagic hemispheres were analyzed by FACS. Neutrophils and macrophages were gated according to the fluorescence intensity of CD45 and CD11b staining. CD45+ CD11b+ cells were gated according to the fluorescence intensity of Ly6G, and CD45+ CD11b- cells were gated and further analyzed for the expression of CD3 and CD19 (Fig. 6C). We observed fewer macrophages (CD45+ CD11b+ Ly6G-) and T cells (CD45+ CD11b- CD3+) in old CCL12^-/-^ mice than in WT mice after ICH, but more macrophages and T cells in CCL12-treated mice than in WT mice (Fig. 6D and Supplementary Fig. 4B and C). In addition, no significant differences were observed in the number of neutrophils (CD45+ CD11b+ Ly6G+) recruited to the perihematomal brain (Fig. 6D and Supplementary Fig. 4A). Thus, these results showed that CCL12 can recruit macrophages and T cells to the damaged brain after ICH.

### CCL12 antibody treatment improves the outcomes of mice after ICH

The above data showed that CCL12 is a detrimental circulating factor that can aggravate brain injury at the cellular and molecular levels after ICH. Next, we investigated whether targeting CCL12 improved the outcomes of old ICH mice. After administering an anti-CCL12 neutralizing antibody via intraperitoneal injection, we observed that the higher NDS and survival rates following ICH (Fig. 7A, B), as well as the BWC in old ICH mice (Fig. 7C), were significantly rescued. Taken together, these data indicate that targeting CCL12 for reduction could improve the outcomes of mice after ICH.

## DISCUSSION

In the current study, we elucidated the roles and mechanisms of CCL12 in aggravating age-related brain injury after ICH. As reported previously, old mice have worse outcomes than young mice after ICH [13]. Furthermore, we observed that the brain and plasma CCL12 levels were markedly upregulated in old mice compared with young mice regardless of ICH, and circulating CCL12 was demonstrated to enter the brain, where it initiated proinflammatory responses. In addition, the results of CCL12 deficiency and supplementation experiments showed that CCL12 aggravates ICH-induced brain injury, which may be correlated with the recruitment of macrophages and T lymphocytes by brain CCL12. Finally, the systemic administration of an anti-CCL12 antibody significantly rescued ICH-induced brain injury in mice. Thus, the results of our study provide evidence that CCL12 may be a potential therapeutic target for the treatment of ICH.

Although remarkable progress has been made in the study of the pathophysiological mechanism of ICH in recent years, thousands of therapeutic drugs that are effective in animal models have been shown to be ineffective when translated into clinical translational studies [24-26]. One of the most important reasons for these outcomes is that most of the basic research subjects are young experimental animals, while the majority of ICH patients are middle-aged or elderly individuals. Therefore, in our study, we used old mice as our primary experimental subjects.

Aging is considered to be an irreversible physiological phenomenon that leads to dysfunction and the aggravation of disease in elderly patients [27, 28]. Many previous studies have demonstrated the protective effects of plasma and its circulating factors from younger individuals on improving brain function. Previously, our group and other research teams also showed that plasma from young rodents could significantly ameliorate acute brain injury in older ICH rodents. In addition to the increased levels of protective factors present in young plasma, inhibiting or targeting the detrimental factors present at elevated levels in plasma from older individuals may also be protective for the brain. For example, targeting CCL11[7] and β2-microglobulin [8] has also been shown to improve brain function. Therefore, in this study, we analyzed the roles of CCL12, a factor that becomes elevated during aging, in influencing acute brain injury after ICH. Our results showed that enhanced circulating CCL12 levels indeed exacerbated acute brain injury in aging ICH mice. However, we still do not know whether the circulating concentration of CCL12 increases with age, and this should be investigated in the future to provide more information regarding the aging process.

Recent studies have shown that the infiltration of peripheral immune cells, such as macrophages, nature killer cells and T lymphocytes, can exacerbate brain injuries after a stroke [29, 30], indicating that inhibiting the infiltration of these peripheral immune cells may be an effective treatment for acute brain injury caused by ICH. In our study, we observed that the enhanced levels of CCL12 in the brain of old mice could aggravate ICH-induced brain injury, possibly via the recruitment of peripheral immune cells, as shown by the significant reduction in the number of macrophages and T lymphocytes in the perihematomal brain tissues of CCL12^-/-^ mice. Furthermore, neutrophils could not be recruited to the perihematomal brain tissues by CCL12. Although previous studies reported that high levels of CCL12 could also recruit eosinophilic granulocytes and leukocytes to damaged areas [31, 32], we did not detect eosinophilic granulocytes, since they are involved in allergic reactions rather than acute injury. However, whether CCL12 aggravates ICH-induced brain injury after ICH via other mechanisms remains to be explored.

## Conclusions

In summary, the results of our study provide a potential therapeutic target for the treatment of ICH in older individuals, and the target was identified based on an analysis of detrimental factors that become more abundant with age. These results suggest that targeting detrimental circulating factors, such as CCL12, in the elderly may be a new therapeutic approach for the treatment of brain injury after ICH.

## Supplementary Materials

The Supplemenantry data can be found online at: www.aginganddisease.org/EN/10.14336/AD.2019.1229.
