# Supplementary material for: Age-related CCL12 Aggravates Intracerebral Hemorrhage-induced Brain Injury via Recruitment of Macrophages and T Lymphocytes
Source: Aging Dis. 2020 Oct 1;11(5):1103–15. doi: 10.14336/AD.2019.1229 (PMC7505273; doi:10.14336/AD.2019.1229)
Supplement: Supplementary file 1 — The Supplemenantry data can be found online at: www.aginganddisease.org/EN/10.14336/AD.2019.1229. [file AD-11-5-1103-suppl.pdf]

SUPPLEMENTARY DATA

Age-related CCL12 Aggravates Intracerebral Hemorrhage-induced Brain Injury via Recruitment of Macrophages and T Lymphocytes

Jiacheng Huang[1,#], Guoqiang Yang[1,#], Xiaoyi Xiong[1,#], Maolin Wang[2], Junjie Yuan[1], Qin Zhang[1], Changxiong Gong[1], Zhongming Qiu[1], Zhaoyou Meng, Rui Xu[1], Qiong Chen[1], Ru Chen[1], Lexing Xie[1], Qi Xie[1], Wenjie Zi[1], Guohui Jiang[1], Yu Zhou[1,*], Qingwu Yang[1,*]

© 2019. Huang J et al. Published online at http://www.aginganddisease.org/EN/10.14336/AD.2019.1229

SUPPLEMENTARY DATA

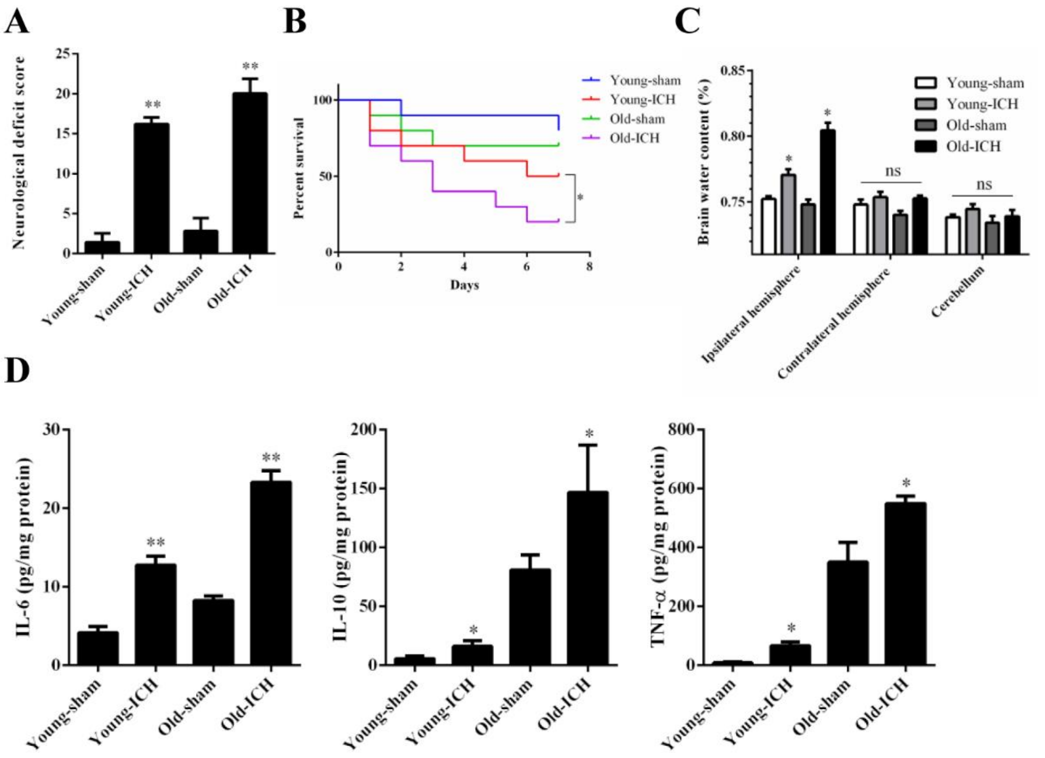

Supplementary Figure 1. Old mice have a poorer prognosis than young mice after ICH. (A) NDS of young and old mice after ICH (n = 6). (B) Mortality rate of young and old mice after ICH (n = 20). (C) BWC in the brain tissues around the hematoma (n = 6). (D) IL-6, IL-10 and TNF-α protein levels in young and old mice detected after ICH by ELISA (n = 6). The bar graphs show the means ± SDs. *P*-values were determined by the ANOVA test. **P* < 0.05; ***P* < 0.01; ns, not significant.

© 2019. Huang J et al. Published online at http://www.aginganddisease.org/EN/10.14336/AD.2019.1229

SUPPLEMENTARY DATA

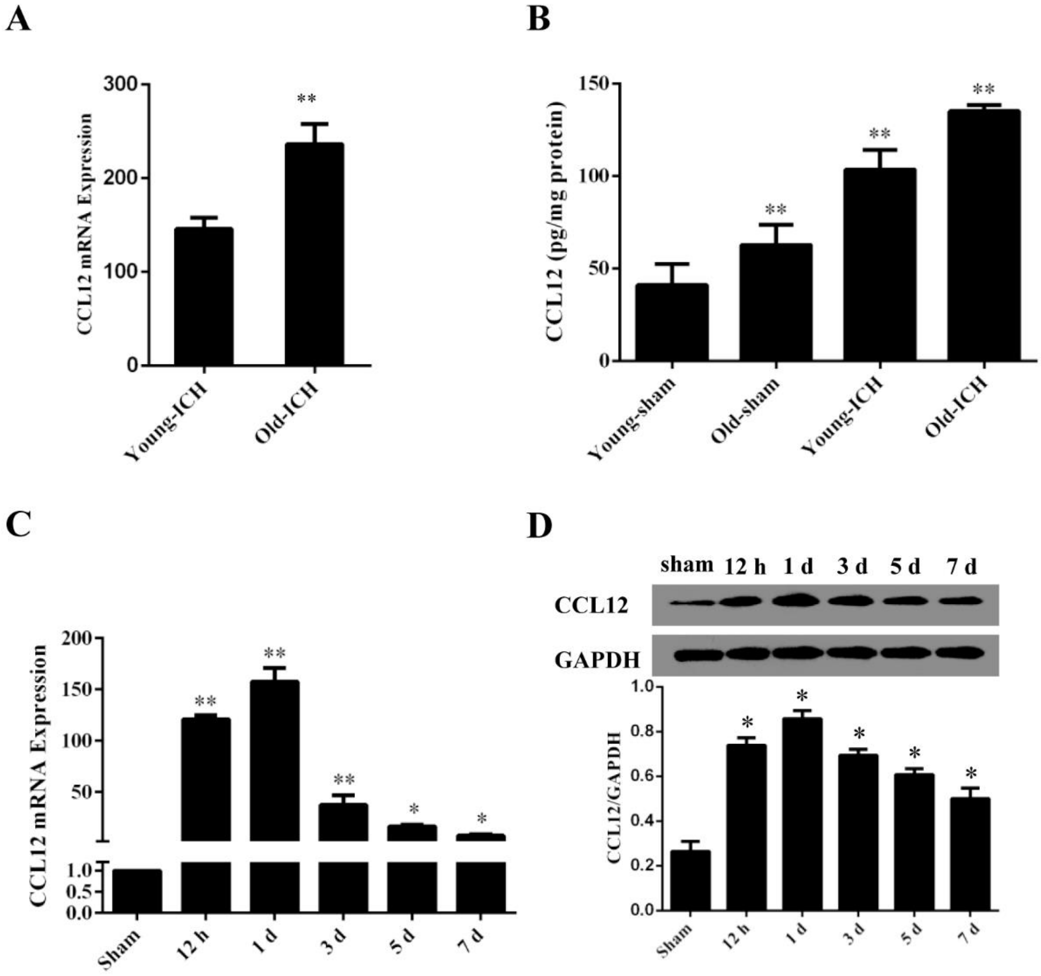

Supplementary Figure 2. CCL12 is upregulated in old mice after ICH. (A) CCL12 mRNA expression in the brain tissues of young and old mice after ICH, as detected by real-time PCR. (B) Plasma CCL12 protein levels in young and old mice in the ICH and sham groups, as detected by ELISA. (C) CCL12 mRNA expression was detected at 12 h, 1 d, 3 d, 5 d and 7 d in ICH and sham-treated mice by real-time PCR. (D) CCL12 protein levels in the perihematomal brain tissues were detected at 12 h, 1 d, 3 d, 5 d and 7 d in ICH and sham group mice, as detected by western blot. The bar graphs show the means ± SDs. *P*-values were determined by ANOVA. **P* < 0.05; ***P* < 0.01; ns, not significant.

© 2019. Huang J et al. Published online at http://www.aginganddisease.org/EN/10.14336/AD.2019.1229

SUPPLEMENTARY DATA

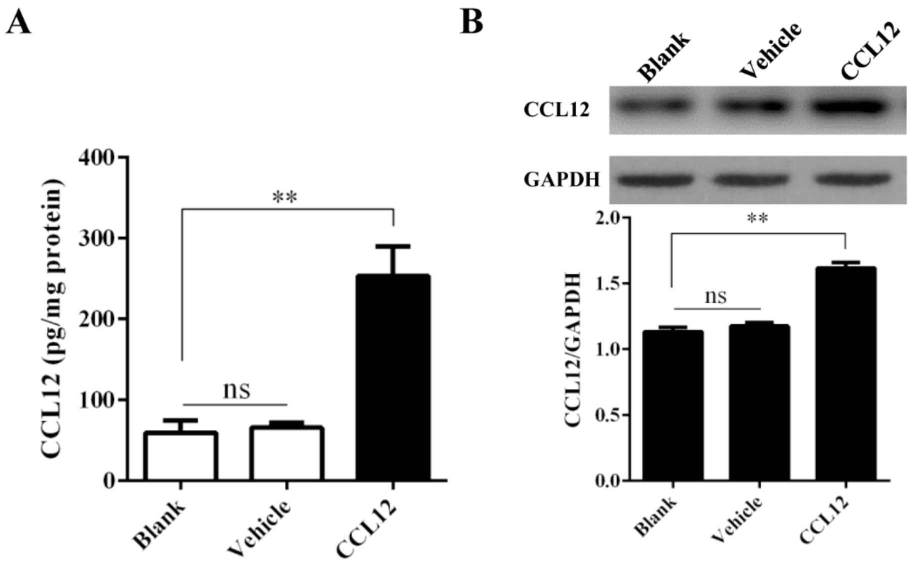

Supplementary Figure 3. CCL12 protein levels of CCL12-treated mice. (A) CCL12 protein levels detected by ELISA before and after CCL12 or vehicle administration. (B) CCL12 protein levels detected by western blot before and after CCL12 or vehicle administration. The bar graphs show the means ± SDs. *P*-values were determined by ANOVA. **P* < 0.05; ***P* < 0.01; ns, not significant.

© 2019. Huang J et al. Published online at http://www.aginganddisease.org/EN/10.14336/AD.2019.1229

SUPPLEMENTARY DATA

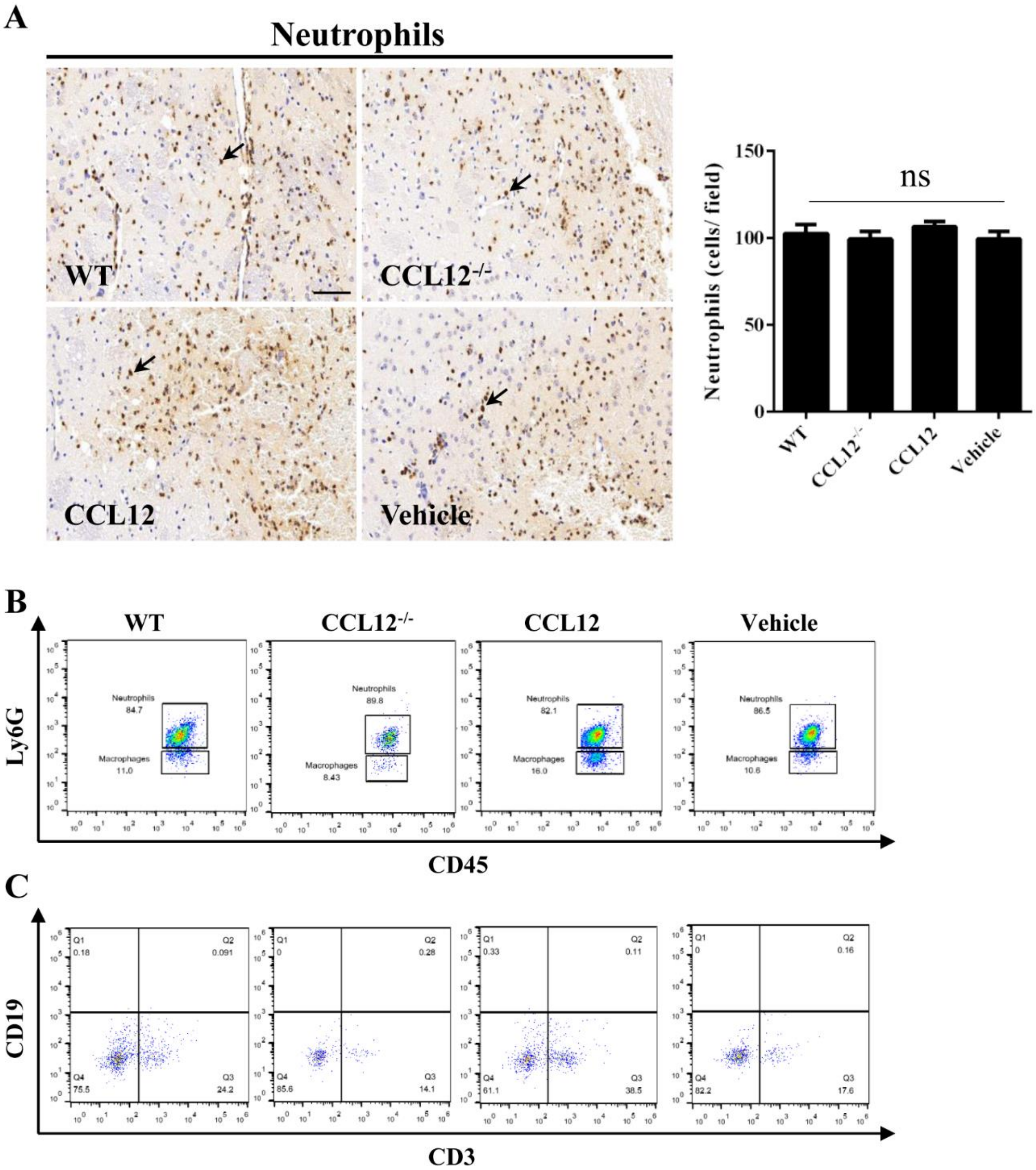

Supplementary Figure 4. The number of neutrophils, CD3+ T cells and macrophages in different groups. (A) Immunohistochemical staining and quantification of positive cells in the perihematomal brain tissues derived from old WT, old CCL12$^{-/-}$, CCL12-treated and vehicle-treated mice after ICH surgery. (B) The number of neutrophils and macrophages in the perihematomal brain tissues derived from old WT, old CCL12$^{-/-}$, CCL12-treated and vehicle-treated mice after ICH surgery detected by FACS. (C) The number of CD3+ T cells in the perihematomal brain tissues derived from old WT, old CCL12$^{-/-}$, CCL12-treated and vehicle-treated mice after ICH surgery detected by FACS. Scale bars, 100 μm. The bar graphs show the means ± SDs. n = 5. *P*-values were determined by ANOVA. **P* < 0.05; ***P* < 0.01; ns, not significant.

© 2019. Huang J et al. Published online at http://www.aginganddisease.org/EN/10.14336/AD.2019.1229
